# Epigenetic editing alleviates Angelman syndrome phenotype in mice by unsilencing paternal Ube3a

**DOI:** 10.1038/s41421-024-00727-3

**Published:** 2024-09-17

**Authors:** Yajing Liu, Sensen Lou, Jinhui Li, Yuanhua Liu, Shisheng Huang, Yu Wei, Jikai Liu, Ruimin Lv, Junjie Tang, Zhixin Shen, Yidi Sun, Xingxu Huang, Zhiqi Xiong, Hui Yang, Changyang Zhou

**Affiliations:** 1 School of Life Science and Technology, Shanghai Tech University, Shanghai, China; 2 Institute of Neuroscience, State Key Laboratory of Neuroscience, Key Laboratory of Primate Neurobiology, CAS Center for Excellence in Brain Science and Intelligence Technology, Shanghai Research Center for Brain Science and Brain-Inspired Intelligence, Shanghai Institutes for Biological Sciences, Chinese Academy of Sciences, Shanghai, China; 3 East China University of Science and Technology, Shanghai, China

**Keywords:** Epigenetics, Epigenetics analysis

Dear Editor,

Angelman syndrome (AS) is characterized by severe neurodevelopmental disorders caused by abnormalities in the maternally inherited *UBE3A* gene, leading to a complete loss of UBE3A specifically in neurons, where the intact paternal *UBE3A* is silenced by an endogenous antisense long-noncoding RNA known as *UBE3A-ATS*. The expression of *UBE3A-ATS* is regulated by the imprinting center *Snrpn* (*Snrpn*-IC)^1^, which is highly methylated in the maternal but not paternal allele. Recent studies using small-molecule inhibitior^2^, RNA targeting^3–5^, artificial transcription factor^6,7^, and CRISPR/Cas9-mediated AAV vector integration^8^ have shown that silencing of *UBE3A-ATS* was effective in allowing paternal *UBE3A* expression and alleviating AS phenotypes in mice. Another potential approach to silencing *UBE3A-ATS* is to introduce DNA methylation through epigenetic editing at the *Snrpn*-IC locus. Here we report that efficient DNA methylation on *Snrpn*-IC can be achieved by catalytically inactive Cas9 (dCas9) fused with peptide epitopes that recruit catalytic domains of two DNA methyltransferases, leading to the unsilencing of paternal *Ube3a* via inhibition of *Ube3a-ATS* expression in mouse neurons (Fig. 1a). By generating AS transgenic mice carrying this epigenetic editing system, the expression of paternal *Ube3a* was markedly increased and AS phenotypes in mice were alleviated. Taken together, these results demonstrate that epigenetic editing can unsilence paternally inherited Ube3a without causing DNA sequence break, suggesting a potential therapeutic approach for AS treatment.

**Fig. 1:**
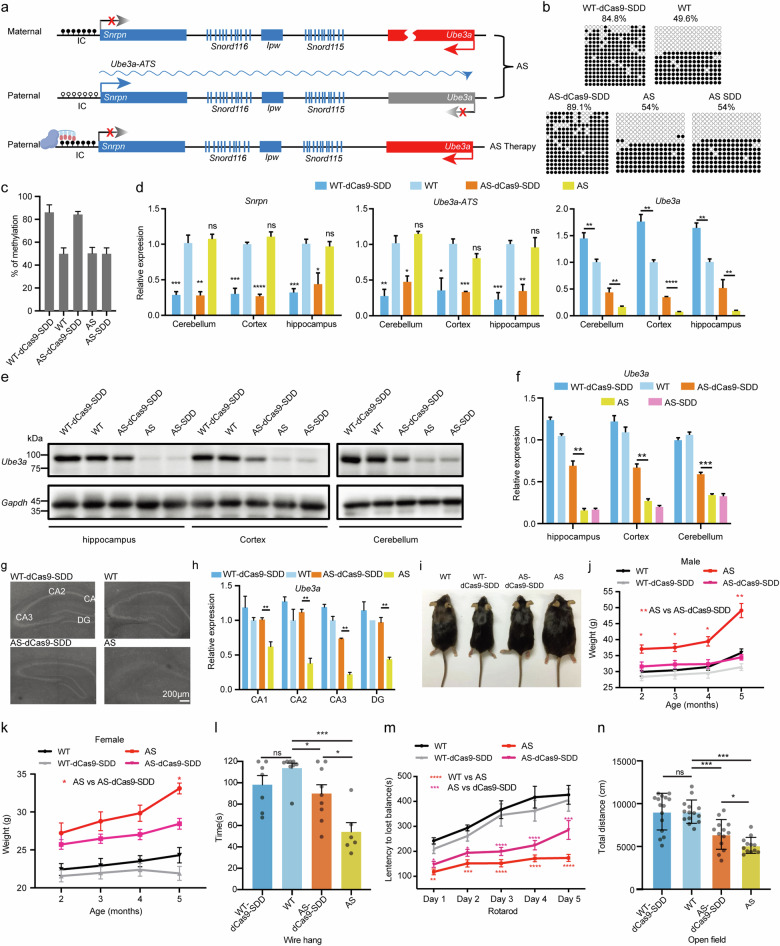
Recovery of paternal Ube3a expression and behavioral alleviation in AS mice via targeted *Snrpn*-IC methylation by the dCas9-SDD system. **a** Diagram of the epigenetic editing-mediated AS therapy. The dCas9-SDD system can introduce methylation at the *Snrpn*-IC and thus inhibit the expression of paternal *Ube3a-ATS*, leading to restored expression of *Ube3a*. **b** The methylation status at *Snrpn*-IC locus of WT-dCas9-SDD, AS-dCas9-SDD, WT, AS-SDD, and AS mice littermates. **c** Quantitative analysis of methylation levels at *Snrpn*-IC locus of WT-dCas9-SDD, AS-dCas9-SDD, WT, AS-SDD, and AS mice. **d** The expression of *Snrpn*, *Ube3a-ATS* and *Ube3a* in cerebellum, cortex and hippocampus among WT-dCas9-SDD, AS-dCas9-SDD, WT, and AS mice. **e** The protein expression level of UBE3A in the indicated brain regions by western blotting assay in WT-dCas9-SDD, AS-dCas9-SDD, WT, and AS mice. **f** Comparison of protein expression levels of UBE3A in the indicated brain regions between the AS-dCas9-SDD and AS mice. **g** The immunofluorescence for UBE3A in the hippocampus of WT-dCas9-SDD, AS-dCas9-SDD, WT, and AS mice. **h** The quantitative expression levels of *Ube3a* by in situ hybridization in the subregions of hippocampus between AS-dCas9-SDD and AS mice. *n* = 3 for each group. **i**–**k** Comparison of body weight of male (**j**) and female (**k**) WT-dCas9-SDD, AS-dCas9-SDD, WT, and AS mice at 2–5 months old. **l** Comparison of the hanging time on the wire of the WT-dCas9-SDD, AS-dCas9-SDD, WT, and AS mice. WT, *n* = 8; WT-dCas9-SDD, *n* = 7; AS, *n* = 6; AS-dCas9-SDD, *n* = 9. **m** The staying time on the rotating rod at day 1 to day 5 of WT-dCas9-SDD, AS-dCas9-SDD, WT, and AS mice. WT, *n* = 14; WT-dCas9-SDD, *n* = 15; AS, *n* = 12; AS-dCas9-SDD, *n* = 13. **n** The total distance traveled in open filed of WT-dCas9-SDD, AS-dCas9-SDD, WT, and AS mice. WT, *n* = 14; WT-dCas9-SDD, *n* = 15 mice; AS, *n* = 12; AS-dCas9-SDD, *n* = 13. All error bars show the SEMs unless otherwise noted. *P < 0.05, **P < 0.01, ***P < 0.001. *P* values were calculated by Student’s *t*-test.

In this study, we designed an epigenetic editing tool that could effectively and precisely methylate targeted DNA sequence. We constructed a vector (termed “dCas9-SDD”) encoding a fusion complex comprising dCas9 protein and repetitive GCN4 peptide epitopes (“SunTag”)^9^, which allow recruitment of multiple copies of the catalytic domain (CD) of de novo DNA methyltransferase 3A and 3L (DNMT3A, DNMT3L) fused with single-chain variable fragment (scFv) to the peptide epitopes (Supplementary Fig. S1a). For comparison, we also constructed a dCas9 system directly fused with DNMT3A’s CD, termed “dCas9-D”, without the Suntag pepetide epitopes.

We first tested the efficiency of dCas9-SDD for targeted DNA methylation by transfecting HEK293T cells with dCas9-SDD construct together with sgRNAs that targeted at the promoter of the transcription factor *BACH2*. The *BACH2* promoter is known to be hypomethylated in HEK293T cells^10^. For comparison, we also transfected a LacZ sgRNA together with either the dCas9-SDD or dCas9-D system into parallel batches of HEK293T cells. We found that the methylation level at the *BACH2* promoter for cells transfected with dCas9-SDD and *BACH2* promoter sgRNA (38.3 ± 1.2%, *n* = 3) was significantly higher than that for cells transfected with dCas9-D and *BACH2* promoter sgRNA (21.5 ± 0.9%, *n* = 3). By constrast, the methylation level in cells transfected with LacZ sgRNA and dCas9-SDD (or dCas9-D) was much lower (2.2 ± 0.7%, *n* = 3) (Supplementary Fig. S1b, c). Notably, the dCas9-SDD system methylated most of the CpG sites at the targeted locus adjacent to the sgRNA binding site, a coverage wider than that of the dCas9-D system (Supplementary Fig. S1b). We found that methylation level of *BACH2* promoter induced by dCas9-SDD was stably maintained for at least 30 days after transfection in HEK293T cells (Supplementary Fig. S1d, e). Analysis of single-cell clones further demonstrated that this high methylation level was maintained for up to 4 months (Supplementary Fig. S1f). In the latter analysis, we observed heterogeneity of the methylated sites at the targeted locus among different cells, even for those in the same clone. This may be attributed to the stochastic activity of endogeneous DNA methyltransferase DNMT1^11^.

We next examined whether the dCas9-SDD system could introduce targeted methylation at the *Snrpn*-IC locus. The sgRNAs targeting *Snrpn*-IC together with the dCas9-SDD vector were transfected into N2a cells and mouse embryonic stem cells (mESCs). We found that the transfected cells showed much higher methylation level than non-transfected wild-type (WT) cells (Supplementary Fig. S2a, b). Additionally, *Snrpn* and *Ube3a-ATS* expression was much lower and *Ube3a* expression was higher in cells that were transfected with dCas9-SDD, as compared with those in WT cells or cells treated with the mutated version of dCas9-SDD (Supplementary Fig. S2c). Furthermore, the reduced expression of *Snrpn* and *Ube3a-ATS* as well as the upregulation of *Ube3a* persisted for at least 30 days in N2a cells (Supplementary Fig. S2d). Finally, we found that lentivirus infection of cultured mouse embryonic neurons with dCas9-SDD (see Supplementary Methods) also resulted in a hypermethylated state of the *Snrpn*-IC, together with the reduced *Snrpn* and *Ube3a-ATS* expression and increased *Ube3a* expression (Supplementary Fig. S2e-g), consistent with the results on cultured N2a cells. Moreover, we tested the long-term effects of epigenetic editing by delivering dCas9-SDD mRNA and sgRNA via lipid nanoparticles (LNPs) in N2a cell culture. This approach resulted in a reduction in *Snrpn* gene expression by >90% for up to ten days post transfection, along with upregulation of *Ube3a* gene expression compared to the control group (Supplementary Fig. S2h). Meanwhile, we performed quantitative PCR (qPCR) and western blotting assay to determine the half-life of dCas9-SDD mRNA and protern in N2a cells. We found that dCas9-SDD mRNA was detectable for no more than 48 h, and the protein for no more than 72 h after treatment (Supplementary Fig. S2i, j). Together, these data indicated that targeted DNA methylation of the *Snrpn*-IC directly led to *Ube3a-ATS* downregulation and consequently unsilencing of Ube3a in mouse neurons.

We further examined the potential off-target effect of our dCas9-SDD system by measuring the methylation level of CpG islands at 15 potential sgRNA-dependent off-target sites. We found only 2 out of 15 sites showed elevated methylation (Supplementary Fig. S3). Such low sgRNA-dependent off-target effect is consistent with the previous finding using Suntag-DNMT3A system. Additionally, we performed high-throughput bisulfite sequencing to examine the off-target methylation of CpGs at the whole-genome level. Through a systematic comparison of methylation levels in cells transfected with dCas9-SDD or SDD, and untransfected WT cells, we found that methylation levels were not significantly different among these three groups of cells at all CpG sites, except for *Snrpn*, which was hypermethylated in the dCas9-SDD group as expected (Supplementary Fig. S4a). Futhermore, We predicted 346 sgRNA-dependent off-target sites (Supplementary Table S2) with target sequences that have up to five mismatches. We analyzed the expression levels of these sites-associated genes in our RNA-seq data, and observed no significant changes between the dCas9-SDD and control group, including the two sites with methylation changes (Supplementary Fig. S4b). Thus, our dCas9-SDD system induced very few sgRNA-dependent and sgRNA-independent off-target DNA methylation.

Given the high efficiency of the dCas-SDD system in introducing DNA methylation in cultured cells, we further explored whether the targeted *Snrpn*-IC methylation could reduce *UBE3A-ATS* expression sufficiently for recovering the paternal UBE3A expression in the AS mouse model. We first generated a transgenic mouse line (*Ube3a*^*p+/m+, dCas9-SDD*^) by integrating the dCas-SDD system into the genome of WT mice (*Ube3a*^*p+/m+*^) using the PiggyBac transposon system. By crossing these mice with female mice deficient in paternal *Ube3a* (*Ube3a*^*p-/m+*^), we generated AS transgenic mice carrying the dCas-SDD system (*Ube3a*^*p+/m-, dCas9-SDD*^ or “AS-dCas-SDD” mice). At the same time, we have obtained three other genotypes for comparison: *Ube3a*^*p+/m+, dCas9-SDD*^ (“WT-dCas9-SDD”), *Ube3a*^*p+/m-, SD*^ (“AS-SDD”) and *Ube3a*^*p+/m-*^(“AS”) (Supplementary Fig. S5a). Consistent with results from cultured cells, we found significant elevation of methylation at the *Snrpn*-IC locus in WT-dCas9-SDD mice (84.8%) and AS-dCas9-SDD mice (89.1%), as compared to that in WT (49.6%), AS-SDD (54%), and AS (54%) littermates (Fig. 1b, c). Moreover, the expression levels of *Snrpn* and *Ube3a-ATS* transcripts were markedly reduced, accompanied by significant elevation of *Ube3a* mRNA expression in WT-dCas9-SDD and AS-dCas9-SDD groups (with targeted DNA methylation), as compared to the WT and AS group (Fig. 1d). Furthermore, the levels of UBE3A protein in the hippocampus, cerebral cortex, and cerebellum of AS-dCas9-SDD mice were also restored to ~80%, 76%, and 70% of the WT level, respectively (Fig. 1e, f). Consistently, immunofluorescence staining of the hippocampus of AS-dCas9-SDD mice showed an elevated expression of UBE3A protein (Fig. 1g, h). To examine the effect of this epigenetic editing system on global gene expression pattern, we compared the transcriptomic patterns between WT-dCas9-SDD and WT mice, as well as between AS-dCas9-SDD and AS mice. We found that the expression levels of the detected genes were similar between the respective groups, except for the targeted gene *Snrpn*, which was downregulated in the dCas9-SDD groups (Supplementary Fig. S6a). Furthermore, qPCR analysis revealed that the expression levels of six other genes sharing the same precursor transcript as *Ube3a-ATS*, as well as five other long genes (*Nrxn3*, 1612 kb; *Astn2*, 1,024 kb; *Pcdh15*, 828 kb; *Csmd1*, 1,643 kb; *Il1rapl1*, 1,368 kb), were not affected by the dCas9-SSD system, whereas topotecan, which impairs transcription elongation, significantly inhibited their expression^2,12^ (Supplementary Fig. S6b–d). Thus, targeted DNA methylation of *Snrpn*-IC can directly reduce *Ube3a-ATS* expression and largely restore UBE3A protein level in AS mice to those of WT mice, without causing detectable off-target effects.

To examine whether the AS phenotypes could be prevented by targeted DNA methylation, we compared the bodyweight of WT, WT-dCas9-SDD, AS, and AS-dCas9-SDD mice (Fig. 1i). The obesity of AS mice is known to occur mostly in young and adult mice^5^. We found that this obesity phenotype was significantly alleviated in AS-dCas9-SDD male mice during 2–5 months after birth, to a level similar to that of WT and WT-dCas9-SDD male mice (Fig. 1j). By contrast, even though AS-dCas9-SDD female mice became heavier than female WT mice, they were notably lighter than AS female mice (Fig. 1k). We note that there was no overweight in WT-dCas9-SDD mice (Fig. 1i, j), suggesting that the downregulation of *Snrpn* gene by dCas9-SDD did not reach the *Snrpn* deficiency associated with Prader-Willi Syndrome, which usually causes obesity^13^.

The behavioral phenotypes of transgenic mice were further evaluated by wire hanging, rotarod running, and open-field test that measure their muscle balance, motor learning, exploratory activity, respectively (Supplementary Fig. S5b). In the wire hanging test, we found that, on average, AS-dCas9-SDD mice remained on the wire siginicantly longer than AS mice (Fig. 1l). In comparison, the average duration was not significantly different between WT-dCas9-SDD and WT mice (Fig. 1l), suggesting that *Snrpn*-IC methylation did not affect movement and balance ability of the transgenic mice. In the rotarod running tests, we found that the average duration of the mouse staying on the rotarod was siginicantly longer for AS-dCas9-SDD mice than AS mice at each training day, whereas that of WT-dCas9-SDD mice and WT mice was similar (Fig. 1m), indicating significant increase of motor learning ability of AS-dCas9-SDD mice. Finally, the AS-dCas9-SDD mice also demonstrated elevated exploratory activity in the open-field test, showing longer distances traveled in the open field than AS mice, whereas the exploratory activity of WT-dCas9-SDD mice was similar to that of WT mice (Fig. 1n). Taken together, these analyses revealed that the behavioral phenotypes of AS mice were significantly alleviated by *Snrpn*-IC-targeted DNA methylation using dCas9-SDD system.

The DNA methylation-mediated silencing of imprinting genes was highly conserved from mice to humans^14^. Here we have demonstrated that targeted DNA methylation could inhibit the expression of the silencing element *Ube3a-ATS*, causing unsilencing of paternal Ube3a in vitro and in vivo. In developing potential therapeutic approaches for AS treatment, this targeted DNA methylation-mediated regulation of *Ube3a-ATS* can achieve precise control of *Ube3a* gene expression, preventing Ube3a overexpression that could cause severe disorders^15^. Silencing *UBE3A-ATS* can also be achieved by CRISPR/Cas9-mediated AAV vector integration^8^, although undesirable mutations or indels could be introduced by endogenous DNA repair machinery associated with direct gene editing. Compared to traditional gene editing tools, epigenetic tools offer the advantage of reversible regulation without altering the DNA sequence. Furthermore, compared to Krab-based transcriptional repression systems, DNA methylation editing tools exhibit better durability with transient expression^7^, allowing delivery through systems like LNP and virus-like particles. In contrast, Krab-based systems require continuous expression via AAV to maintain gene suppression. Thus, DNA editing systems provide more flexible delivery options for future disease treatments. In this work, we introduced epigentic editing via transgenic approach in the embryos, with the editing controlled by neuron-specific synapsin promoter, thus allowed targeted DNA methylation in all neurons. This approach is particularly suitable for modifying AS phenotype, since silencing of paternal *Ube3a* allele was found only in neurons^1^. Future development of delivery system for epigenetic editing system targeting DNA methylation at *Snrpn*-IC may offer an effective approach for the treatment of AS patients.

## Supplementary information

Supplementary Information

Supplementary Information Table S2

## Data Availability

All the sequencing data were deposited in NCBI Sequence Read Archive (SRA) under the project accession numbers PRJNA842138 and PRJNA842173.
