# Supplementary material for: Epigenetic editing alleviates Angelman syndrome phenotype in mice by unsilencing paternal Ube3a
Source: Cell Discov. 2024 Sep 17;10:97. doi: 10.1038/s41421-024-00727-3 (PMC11405779; doi:10.1038/s41421-024-00727-3)
Supplement: Supplementary file 1 — Supplementary Information [file 41421_2024_727_MOESM1_ESM.pdf]

SUPPLEMENTARY INFORMATION

Methods and Materials

Plasmid construction

To construct the dCas9-GCN4 plasmid, First, we synthesized a 10×GCN4 fragment; each GCN4 was connected with GSGSGGSGSGGSGSGSGGSGSGGSGSG linker. Second, the dCas9-GCN4 plasmid was obtained from the C-terminal connected cassette of dCas9(Addgene, #107307) replaced by 10×GCN4 fragment. In order to construct the scFv-DNMT3L (CD)-DNMT3A (CD) plasmid, we amplified DNMT3L (CD) and DNMT3A (CD) fragment from 293T cDNA, and obtained the scFv and GB1 element from pHRdSV40-scFv-GCN4-sfGFP-VP64-GB1-NLS plasmid (Addgene, #60904). SDD plasmid was generated by assembling these fragments with dCas9-GCN4 plasmid backbone. In SDD transgenic mice, the CAG promoter was replaced by the human SYN1 promoter (hSYN1). The oligos used to generate sgRNA plasmids (Supplementary Table S1) were annealed and insert into the BsaI sites of pGL3-U6-sgRNA-PGK-Puro (Addgene, #51133).

Cell culture and transfection

HEK293T (ATCC CRL-3216) and Neuro-2a (N2a) (ATCC HTB-96) cells were cultured in Dulbecco's Modified Eagle's Medium (DMEM) (Hyclone, SH30243.01) supplemented with 10% fetal bovine serum (FBS) (v/v) (Gemini, 900-108) and penicillin and streptomycin (Gibco, 15140122). All cells we used in experiment have been tested to exclude mycoplasma contamination. Cells were seeded on 12-well plates (JET-BIOFIL, TCP010012) and transfected about 14 hours thereafter at approximately 80% density per the manufacturer's protocols (ThermoFisher Scientific, 11668019). dCas9-GCN4-GFP and SDD-mCherry expressing vectors (400 ng for each plasmid) were co-transfected with corresponding sgRNA plasmids (200 ng), and the cells with both GFP and mCherry signal were isolated by FACS 48 hours later. The isolated cells were analyzed for DNA methylation and RNA expression.

Neurons were dissected from 13.5 days (P1) AS embryos as previously described (Kaech S & Banker G (2007) Culturing hippocampal neurons. Nature protocols 1:2406.). The neurons were cultured in Neurobasal medium with 1% B27 and 10mM GlutaMax, and the medium was half refreshed each 3 days. We used multiplicity of infection (MOI) of 10 in primary cultures. For rescue experiments, dCas9-GCN4 and SDD lentivirus were mixed and infected culture neurons at 5 DIV (day in vitro). The neurons were collected for further analysis at 7 days after infection.

Lentivirus packaging

To generate lentivirus to infect TDM system into neurons, we plated HEK293T cells on ten 15-cm tissue culture plates. On each plate, 1×10^6 HEK293T cells were plated in 30 mL of DMEM with 10% FBS, grown overnight, and then transfected with 8 mg of an equimolar mixture of the three third-generation packaging plasmids (pMD2.G #12259, psPAX2 #12260, pMDLg/pRRE #12251) and 8 mg of dCas9-GCN4 or SDD vectors using 50 μL of polyethylenimine (PEI, Polysciences #23966). After 48 hours and 72 hours of incubation, lentivirus was harvested. We filtered the pooled lentivirus through a 0.45-mm PVDF filter (Millipore) to remove any cellular debris.

Mouse lines and breeding strategies

All experimental procedures were approved by the Institutional Animal Care and Use Committee of the Institute of Neuroscience, Chinese Academy of Science and were in according with the Society for Neuroscience guidelines. All of the mice were under the C57BL/6J background and housed on a 12 h light /12 h dark cycle. The day on which the vaginal plug was detected was designated as embryonic day 0.5 (E0.5). Ube3a deletion mice were generated by Jiang and colleagues (Jiang Y-h, et al. (1998) Mutation of the Angelman Ubiquitin Ligase in Mice Causes Increased Cytoplasmic p53 and Deficits of Contextual Learning and Long-Term Potentiation. Neuron 21:799–811). Ube3am-/p+ mice were generated by crossing the Ube3am+/p- heterogenous females to wildtype (WT) males.

To generate transgenic mice with the dCas9-GCN4 or SDD elements, we utilized the PiggyBac system for inserting these elements into the mouse genomic DNA. This process involved collecting zygotes and injecting a mixture containing Super PiggyBac Transposase (PB200PA-1) mRNA along with the dCas9-GCN4 or SDD plasmid. For the production of mice simultaneously expressing both dCas9-GCN4 and SDD, we bred male mice harboring the dCas9-GCN4 element with female mice carrying the SDD element. This crossbreeding resulted in adult offspring equipped with the combined methylation system.

Bisulfite sequencing and analysis

Bisulfite conversion of DNA was established using the EZ DNA Methylation-GoldTM Kit (ZYMO RESEARCH, D5006) following the manufacturer's instructions. The resulting modified DNA was amplified by first round of nested PCR, following a second round using Ex Taq® Hot Start Version DNA Polymerase (Takara, RR006B) and loci specific PCR primers (Supplementary Table S3). The first round of nested PCR was done as follows: 98°C for 5 min; 95°C for 40 s, 48°C for 40 s, 72°C for 1 min; Repeat steps 2-4 35×; 72°C for 5 min; Hold 12°C. The second round of PCR was as follows: 98°C for 5 min; 95°C for 40 s, 55°C for 40 s, 72°C for 1 min; Repeat steps 2-4 35×; 72°C for 5 min; Hold 12°C. The resulting amplified products were gel-purified, sub-cloned into a pMD™19-T Vector (Takara), and sequenced by M13F(-47) 5'-CGCCAGGGTTTTCCCAGTCACGAC-3'. The sequencing date were analyzed on website: http://quma.cdb.riken.jp/.

Quantitative reverse transcription PCR

Total RNA was isolated using TRIzol™ Reagent (Invitrogen, 15596018) according to the manufacturer's instructions. cDNA was synthesized using HiScript® II Q RT SuperMix for qPCR (+g DNA wiper) Kit (Vazyme, R223-01), and quantitative reverse transcription PCR (qRT-PCR) was performed on a QuantStudio™ 7 Flex Real-Time PCR System using ChamQ SYBR qPCR Master Mix (Low ROX Premixed) (Vazyme, Q331-03) and gene-specific primers (Supplementary Table S3). Quantitative analysis was performed employing the ΔΔCT method and the GAPDH as the endogenous control.

Western blot analysis

The mice were sacrificed after anesthesia, and the hippocampus, prefrontal cortex and cerebellum were taken separately on ice, and then lysed in RIPA buffer (Beyotime, P0013B) plus a complete protease inhibitor cocktail (Beyotime, P1005) and PMSF (Beyotime, ST506). Lysates were

centrifuged and supernatants were subjected to SDS-PAGE. Primary antibodies were as follows: Mouse monoclonal anti-E6AP antibody (1:1000, SIGMA, E8655-200UL), mouse monoclonal anti-GAPDH antibody (1:5000, Sigma, G8795); The blots were developed using an Immobilon Western Chemilum HRP Substrate kit (Millipore, WBKLS0100). Protein levels were quantified by densitometry using the ImageJ software.

LNP Formulation and Characterization

For mouse studies, LNPs were formulated following previously described methods with some modifications. Briefly, an ethanolic solution of 1,2-distearoyl-sn-glycero-3-phosphocholine, cholesterol, a PEG lipid, and an ionizable cationic lipid was rapidly mixed with an aqueous solution (pH = 4) containing mRNA and sgRNA in a 1:1 weight ratio using an in-line mixer at a 1:3 ethanol to aqueous phase flow ratio. The N:P ratio between the ionizable lipids and nucleic acids was maintained at 4 to 6 throughout the study.

The resulting LNP formulation was dialyzed overnight against 1× PBS, 0.22 μm sterile filtered, and stored at 4°C. The particle sizes ranged from 60 to 80 nm (Z-Ave, hydrodynamic diameter) with a polydispersity index of less than 0.1, as determined by dynamic light scattering (Malvern NanoZS Zetasizer). RNA encapsulation efficiency in the LNPs was measured using the Quant-iT Ribogreen Assay (Life Technologies).

Immunofluorescence

For immunostaining of the neuronal culture, neurons were fixed in 4% paraformaldehyde and then permeabilized with 0.25% Triton X-100. After blocking with 5% goat serum and 0.1% Triton X-100, cells were incubated with appropriate primary antibodies overnight at 4°C. After washing, cells were incubated with secondary antibodies for 1 h at room temperature. For immunostaining of brain slices, mice were euthanized and transcranial perfused with 4% PFA in PBS. Fixed tissue was sectioned with 30 μm thickness using vibratome (Leica, VT1000S). Sections were blocked with 5% normal goat serum (NGS) in TBST (137 mM NaCl, 20 mM Tris pH 7.6, 0.05% Tween-20) for 1 h and incubated with primary antibodies overnight at 4°C. After three washes in TBST, samples were incubated with secondary antibodies. Fluorescent signals were detected on an Olympus FV1200 confocal microscope by sequential acquisition or on slide scanner (Zeiss, Axio Scan.Z1) and images were processed using ImageJ software.

Primary antibodies were as follows: Mouse monoclonal anti-E6AP antibody (1:1000, SIGMA, E8655-200UL), mouse monoclonal anti-GAPDH antibody (1:5000, Sigma, G8795)

Whole-genome bisulfite sequencing

One hundred nanograms of DNA was used for WGBS libraries preparation using a TruSeq DNA methylation kit according to the manufacturer's instructions (Illumina). Libraries were sequenced using a NextSeq 500 sequencer.

For each WGBS profile, we used trim-galore (0.6.6) to trim adapter and low-quality sequences as the default threshold and aligned the bisulfite-treated reads to the mouse genome (grcm38) and cytosine methylation was called using bismark[1] (bowtie2 was introduced for alignment).

CpG methylation levels at the promoter regions (-500 to +200 bp of Tss) of all the transcripts were calculated and the regions with less than 5 CpGs were filtered for further differential analysis. We identified the significantly methylated genes using limma with the threshold of adjusted p value

less than 0.05.

RNA sequencing

RNA from dCas9-SunTag-DNMT3A-treated samples was extracted using a RNeasy Micro Kit (Qiagen) and quantified using Nanodrop. The TruSeq stranded mRNA library preparation was based on the manufacturer's instructions (Illumina). Libraries were sequencing using a NextSeq 500 sequencer. Paired-end RNA-seq reads were mapped to the human genome (hg19) using TopHat 2.0.10. The fragments per kilobase of exon per million fragments mapped (FPKM) values were calculated using Cufflinks 2.2.1.

Behavioral methods

Six-week-old mice of different genotypes were evaluated for each behavioral test including rotarod test, open field test, elevated plus maze, tail suspension, and fear conditioning test. Observers were blinded to the mouse genotype during all testing. Behavioral tests were performed in the behavioral test core of the Institute of Neuroscience of Chinese Academy of Sciences and analyzed with automated system. All behavioral tests were conducted in the same group of animals. The data were analyzed by EthoVision XT 11.5 software (Noldus). We recorded both female and male mice body weight every month from the age of 8 weeks.

Rotarod test

This test was used to measure motor balance and coordination by placing mice on an accelerating, 3 cm diameter rotating rod (Ugo Basile, Italy) for three trials, with a minimum 15-min interval between each trial. The rotarod was started at 4 rpm and increased to 40 rpm over a period of 5 min. The mean latency to fall off during the three trials was recorded for analysis. Mice from each group were pre-trained for adaptation at the first day and recorded at the subsequent days.

Open-field test

This test was used to measure the exploratory locomotor activity. Each mouse was placed in the center of a transparent plastic chamber (40 cm × 40 cm × 40 cm) and allowed to explore freely for 15min. The testing arena was brightly lit. During each session, their behavior was automatically videotaped and subsequently analyzed using the EthoVision video tracking system.

Tail suspension test

An automated tail suspension system (TAILSUSP-1N96, MED Associates) was used to measure the immobility of suspended mice. The animal's tail was wrapped by adhesive tape at a constant site three quarters of the distance from the base of the tail. The mice were then suspended by passing the suspension hook through a metal chain and the total time of immobility was recorded for analysis automatically.

Statistical analysis

All experiments and data analysis were performed by investigators blind to the mouse genotypes and treatments. All computed parameters were quantified and compared between tested groups unless specified otherwise. No animals were excluded from the analysis. Data were presented as mean ± SEM of the values from at least three independent experiments. The sample size of each experimental group was selected to minimize the number of mice used in the studies while providing sufficient information to report significant and reliable results. No statistical method was used to predetermine sample size. Significance between two means was analyzed using two-sided unpaired

Student's t-test. Comparisons between multiple groups were assessed by one-way ANOVA with Dunnett's post hoc analysis or two-way ANOVA with Bonferroni post hoc test. The nonparametric Mann-Whitney U-test was used in comparisons of < 6 cases. We used Fisher's exact test to calculate P-values comparing two ratios. All statistical analyses were performed using SPSS 15.0 software. $P < 0.05$ was considered statistically significant.

Data availability

The data that support the findings of this study are available from the corresponding author upon reasonable request.

References:

1. Krueger, F. & Andrews, S. R. Bismark: a flexible aligner and methylation caller for Bisulfite-Seq applications. *Bioinformatics* **27**, 1571–1572 (2011).

SUPPLEMENTARY FIGURES

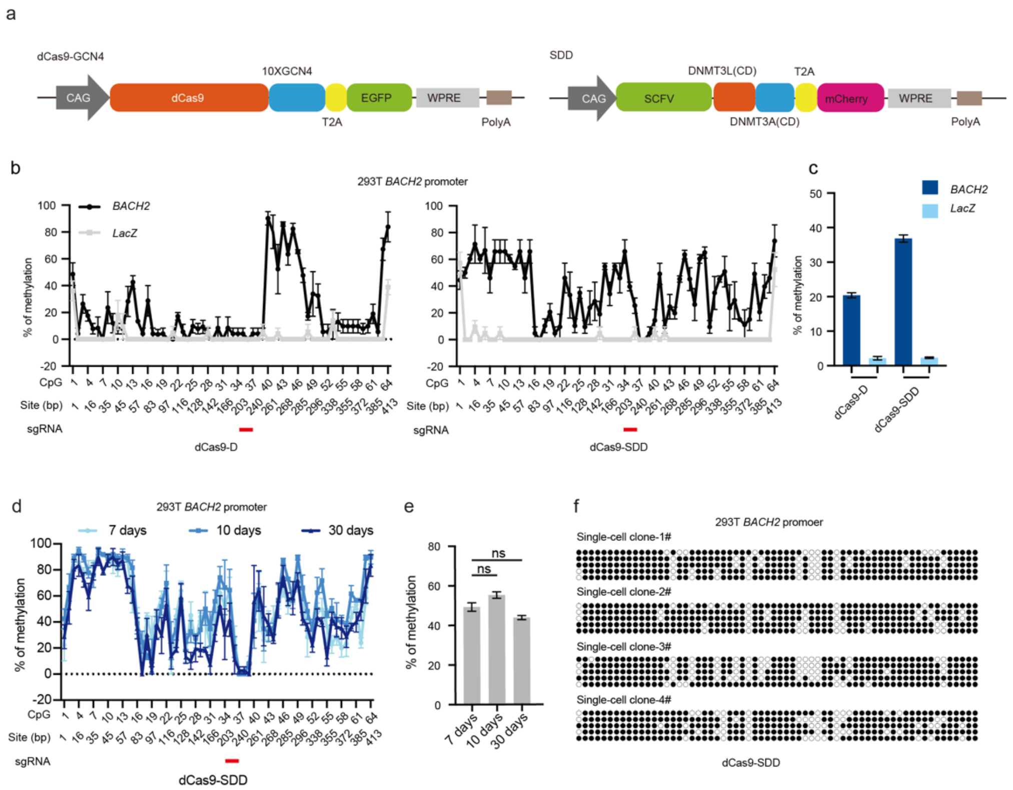

Supplementary Fig. S1 Comparison of methylation efficiency and durability across different mythylation systems.

a, The construct of dCas9-GCN4 and SDD system. **b**, The percentage of cells that were successful methylated on the targeted sites by the dCas9-D or dCas9-SDD system. Shown is the mean percentage ±SEM of two biological replicates. **c**, Comparison of methylation efficiency on the promoter of *BACH2* between dCas9-D and dCas9-SDD system. **d**, The percentage of cells that were successful methylated on the targeted sites at 7, 10 and 30 days post transfection of the dCas9-SDD system. **e**, Comparison of methylation efficiency on the promoter of *BACH2* at 7, 10 and 30 days post transfection of the dCas9-SDD system. **f**, Methylation at the *BASH2* promoter in 4 single clones of HEK293T cells assessed 4 mouths post-transfection. All error bars show the s.e.m. unless otherwise noted.

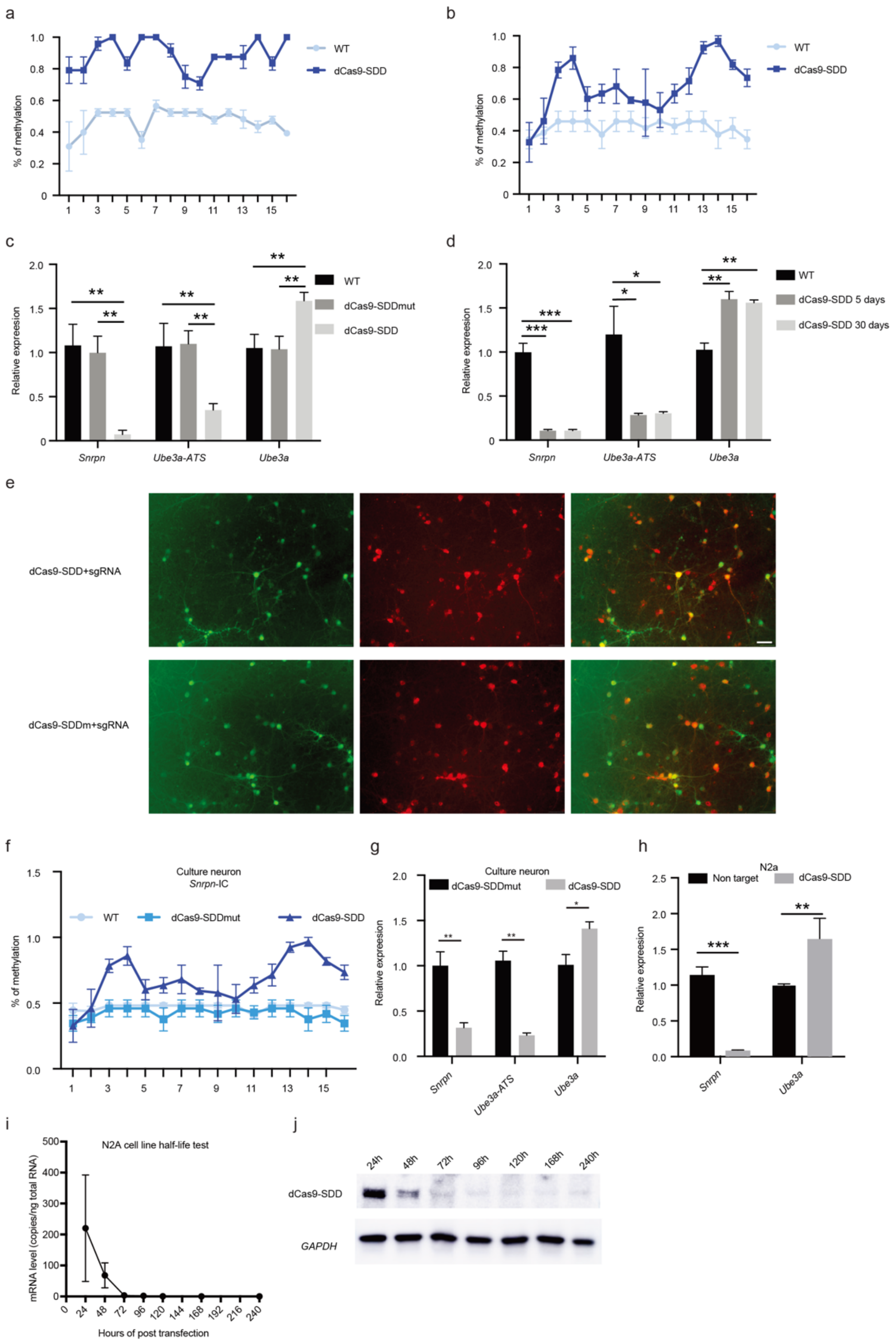

Supplementary Fig. S2 Unsilencing paternal Ube3a by introducing methylation on Snrpn-IC using dCas9-SDD system in culture cells.

a, The methylation level at Sprpn-IC in N2a cells 7 days post-transfection with dCas9-SDD system or wild-type N2a cells. Shown is the mean percentage ±SEM of two biological replicates. **b**, The methylation level at Sprpn-IC in mESCs 7 days post-transfection of the dCas9-SDD system. Shown

is the mean percentage ±SEM of two biological replicates. **c**, Comparison of the expression levels of indicated genes in N2a cells 7 days post-transfection with dCas9-SDD system or dCas9-SDDmut system. **d**, The expression levels of indicated genes in N2a cells at 5 and 30 days post-transfection with dCas9-SDD system. **e**, The co-expression of neurons and dCas9-SDD system. n = 3 for each group. **f**, The percentage of cells that were successful methylated on the *Snrpn-IC* in WT, dCas9-SDD or dCas9-SDDmut transfected culture neurons. Shown is the mean percentage ±SEM of two biological replicates. **g**, Expression of the indicated genes 7 days after transducing culture neurons with dCas9-SDD or dCas9-SDDmut system. n = 3 for each group. **h**, Expression of the *Snrpn* and *Ube3a* gene 10 days after treatment with dCas9-SDD mRNA and *Snrpn* sgRNA or non-target sgRNA delivered by lipid nanoparticles (LNPs). **i**. A time course of dCas9-SDD mRNA level detected by qPCR in N2A cells treated with dCas9-SDD at a dose of 2.5ug/mL. **j**. A time course of dCas9-SDD protein level detected by western blot with anti-Cas9 antibody in N2a cells treated with dCas9-SDD at a dose of 2.5ug/mL. All error bars show the s.e.m. unless otherwise noted. *$P < 0.05$, **$P < 0.05$, ***$P < 0.05$. P values were calculated by Student t-test.

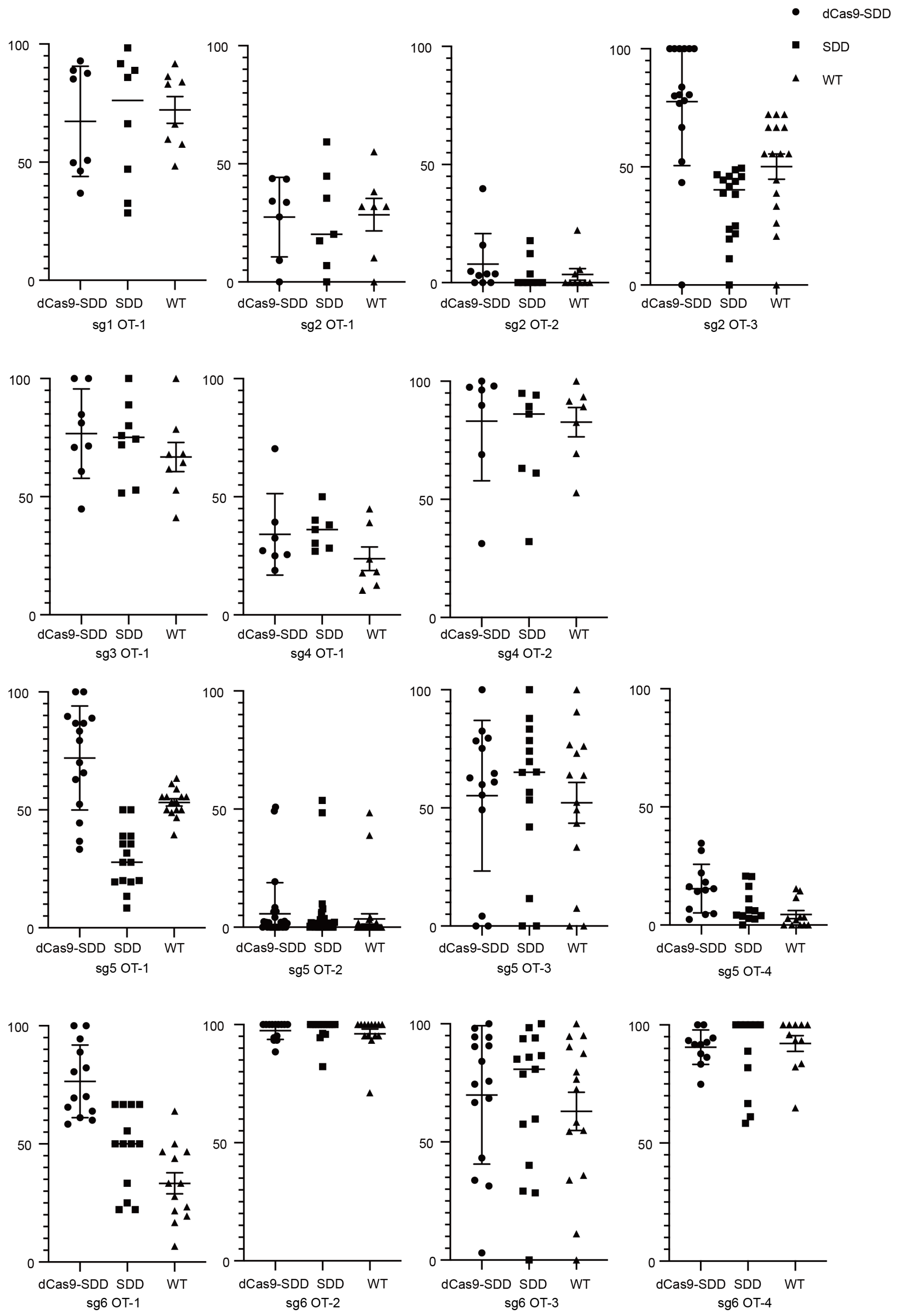

Supplementary Fig. S3 The methylation level at predicted off target sites in HEK293T cells. The methylation levels at 15 predicted sgRNA-dependent off-target sites in HEK293T cells transfected with dCas9-SDD or SDD systems.

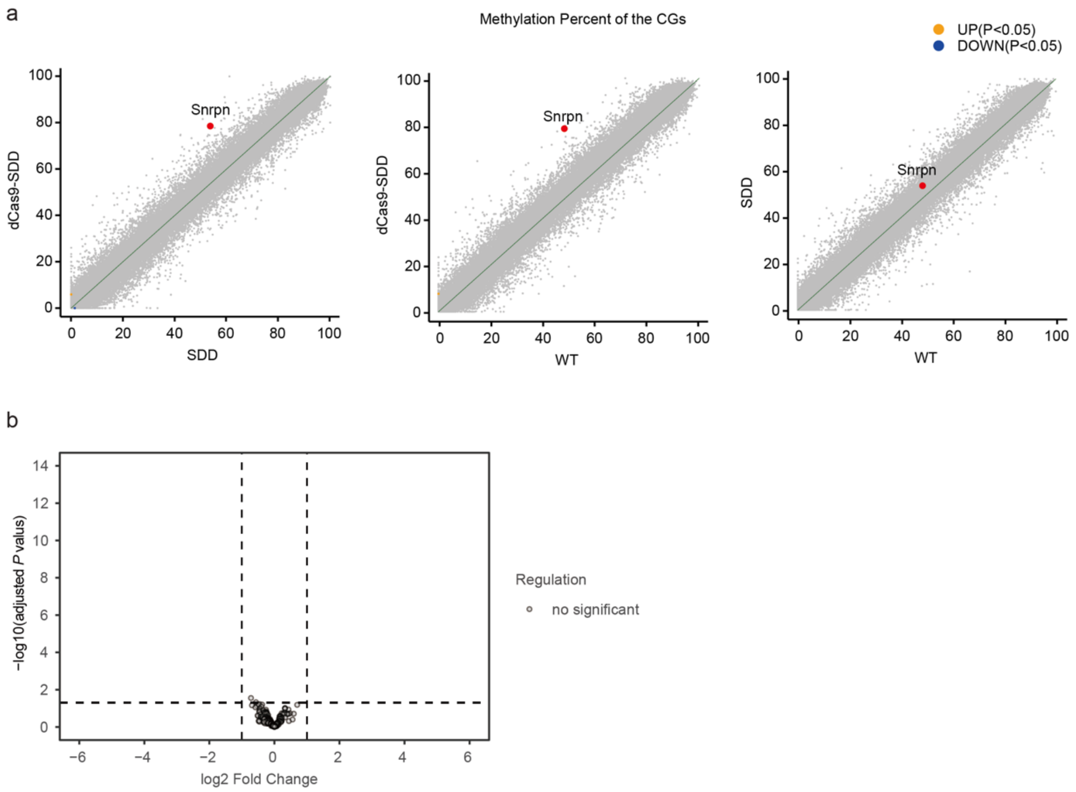

Supplementary Fig. S4 The genome-wide off-target evaluation of dCas9-SDD system by whole genome bisulfate sequencing and RNA-seq.

a, Comparison of the methylation levels of geneme-wide CpG islands in WT HEK293T cells and cells transfected with dCas9-SDD or SDD system. **b**, Volcano plot showing the fold changes and adjusted P values of genes associated with sgRNA-dependent off-target sites between experiment and control groups.

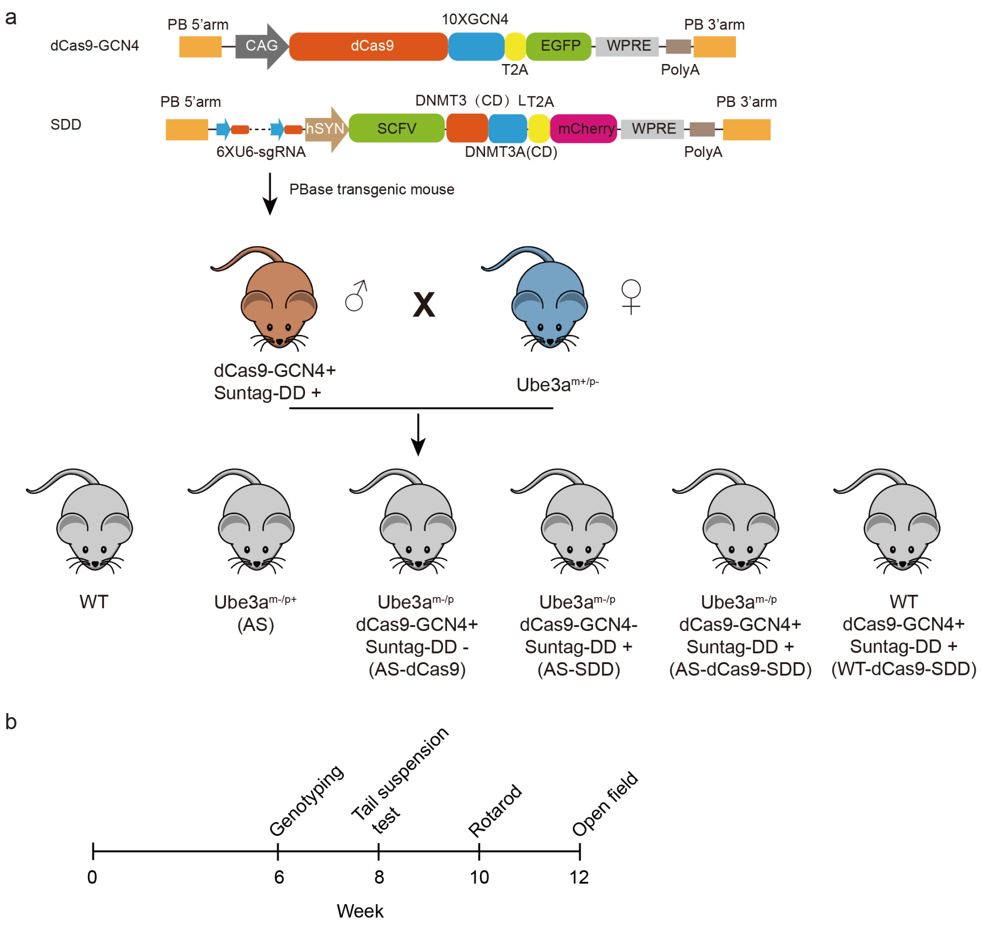

Supplementary Fig. S5 The diagram of generating AS transgenic mice.

a, This diagram illustrates the process of creating the Angelman Syndrome (AS) transgenic mouse line. Initially, a transgenic mouse line (Ube3a$^{p+/m+,\ dCas9\text{-}SDD}$) was generated by integrating the dCas9-SDD system into the genome of wild-type (WT) mice (Ube3a$^{p+/m+}$) using the PiggyBac transposon system. These mice were then crossed with female mice deficient in paternal Ube3a (Ube3a$^{p-/m+}$), resulting in the production of AS transgenic mice carrying the dCas9-SDD system (Ube3a$^{p+/m-,\ dCas9\text{-}SDD}$ or 'AS-dCas-SDD' mice). Concurrently, four other genotypes were obtained for comparative purposes: Ube3a$^{p+/m+,\ dCas9\text{-}SDD}$ ('WT-dCas9-SDD'), Ube3a$^{p+/m-,\ SDD}$ ('AS-SDD'), Ube3a$^{p+/m-,\ dCas9}$ ('AS-dCas9') and Ube3a$^{p+/m-}$ ('AS').

b, Timeline of behavior assays performed on AS transgenic mice.

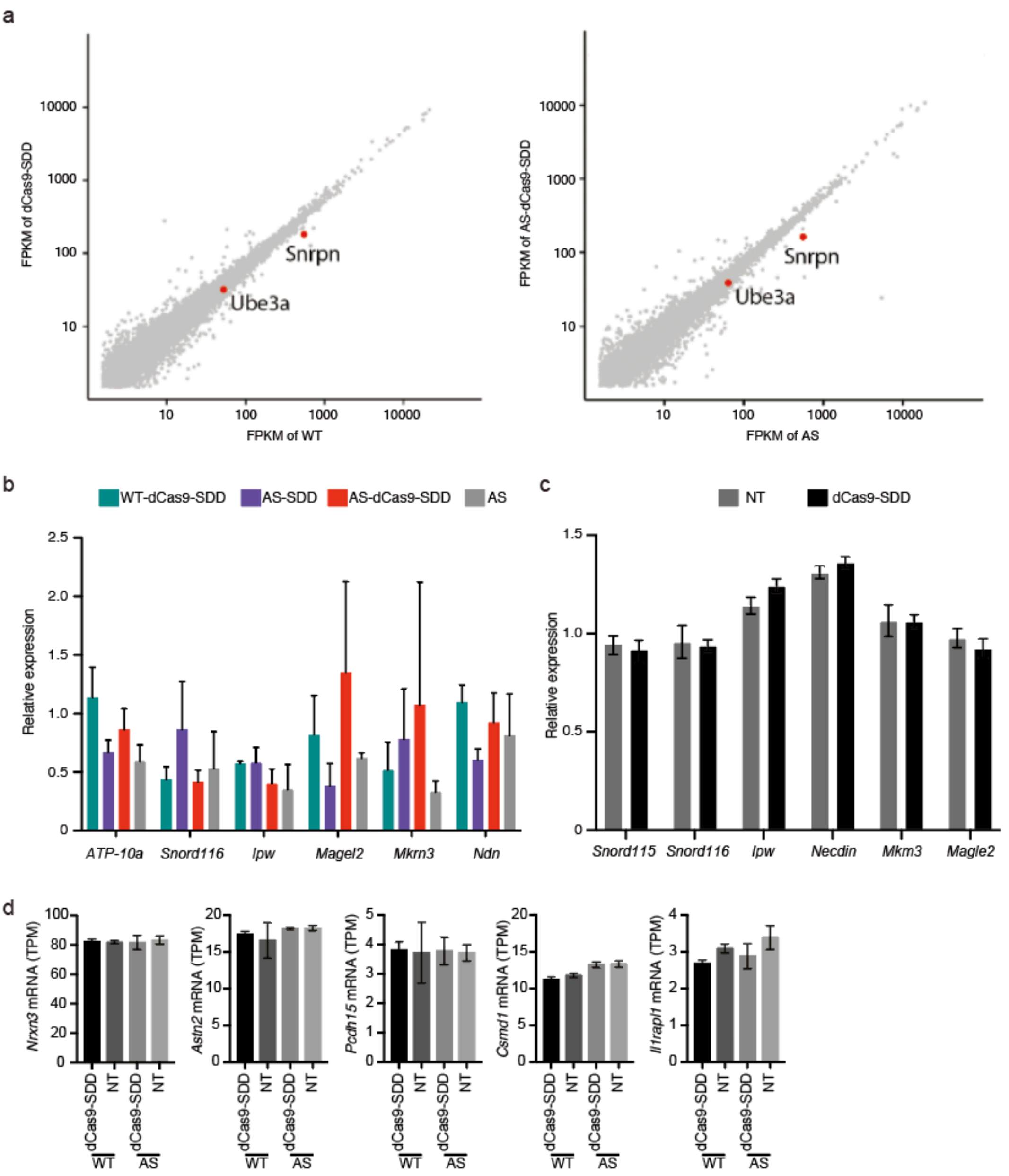

Supplementary Fig. S6 The transcriptome wide off-target evaluation of dCas9-SDD system by RNA-seq.

a, The comparison of transcriptome-wide gene expression levels between WT-dCas9-SDD and WT mice or AS-dCas9-SDD and AS mice. **b**, The expression levels of indicated genes in WT-dCas9-SDD, AS-SDD, AS-dCas9-SDD, and AS mice. n = 3 for each group c, The expression levels if indicated genes 10 days after treatment with dCas9-SDD mRNA and Snrpn sgRNA or non-target sgRNA delivered by lipid nanoparticles (LNPs). **d**,The expression level of indicated genes captured in RNA-seq data in WT-dCas9-SDD, AS-SDD, AS-dCas9-SDD, and AS mice. $n = 3$ for each group. All error bars show the s.e.m. unless otherwise noted. *$P < 0.05$, **$P < 0.05$, ***$P < 0.05$. P values were calculated by Student t-test.

Supplementary Table S1. sgRNA sequences.

Snrpn-sgRNA-1	tttggtagctgccttttggc
Snrpn-sgRNA-2	cgcatgtgcagccattgcct
Snrpn-sgRNA-3	gcgacaaacctgagccattg
Snrpn-sgRNA-4	gaccaaaacattctagattt
Snrpn-sgRNA-5	actccttgggtgtgttagtg
Snrpn-sgRNA-6	tgccgcagcgtgggggctcc

Supplementary Table S3. Primer list

Mus *Atp10a*	Primer-F	GTTGTGCCACATCGAGACTG
	Primer-R	GTGAACGTCAGAGGGTTGAAT
Mus *Snord116*	Primer-F	ggatctatgatgattcccag
	Primer-R	ggacctcagttccgatga
Mus *Snord115*	Primer-F	CTGGGTCAATGATGACAAC
	Primer-R	TTGGGCCTCAGCGTAATCC
Mus *Ipw*	Primer-F	GATGCATTCCTTTTCCTTCA
	Primer-R	TGGTAGAAGAAATGGCACCATC
Mus *Magel2*	Primer-F	ATCCAGTCTCAAGTCATAAGGGC
	Primer-R	CTGCCATGTCAAAGGCGTT
Mus *Mkrn3*	Primer-F	ACAGGTGTGCATACCCCCA
	Primer-R	GCAGGCCCTTCTATGAGCTTC
Mus *Ndn*	Primer-F	GAGGTCCCCGACTGTGAGAT
	Primer-R	TGCAGGATTTTAGGGTCAACATC
Hum *BACH2* (Bisulfite sequencing)	Primer-OF	GGGTYGGGAGGAGGGGGTTTTT
	Primer-IF	AGYGGGGAGATTTGTTGTTG
	Primer-IR	AAAATAAACAAACRAAATAA
	Primer-OR	CATCACATAACAACTCRTTCCCAAC
